# Biochemical and Virulence Characterization of *Vibrio vulnificus* Isolates From Clinical and Environmental Sources

**DOI:** 10.3389/fcimb.2021.637019

**Published:** 2021-02-26

**Authors:** Keri A. Lydon, Thomas Kinsey, Chinh Le, Paul A. Gulig, Jessica L. Jones

**Affiliations:** ^1^ Division of Seafood Science and Technology, Gulf Coast Seafood Laboratory, U.S. Food and Drug Administration, Dauphin Island, AL, United States; ^2^ Department of Molecular Genetics and Microbiology, University of Florida, Gainesville, FL, United States

**Keywords:** *Vibrio*, mouse model, mannitol, virulence, 16S rDNA****, season

## Abstract

*Vibrio vulnificus* is a deadly human pathogen for which infections occur *via* seafood consumption (foodborne) or direct contact with wounds. Virulence is not fully characterized for this organism; however, there is evidence of biochemical and genotypic correlations with virulence potential. In this study, biochemical profiles and virulence genotype, based on 16S rRNA gene (*rrn*) and virulence correlated gene (*vcg*) types, were determined for 30 clinical and 39 oyster isolates. Oyster isolates were more biochemically diverse than the clinical isolates, with four of the 20 tests producing variable (defined as 20–80% of isolates) results. Whereas, for clinical isolates only mannitol fermentation, which has previously been associated with virulence potential, varied among the isolates. Nearly half (43%) of clinical isolates were the more virulent genotype (*rrn*B/*vcg*C); this trend was consistent when only looking at clinical isolates from blood. The majority (64%) of oyster isolates were the less virulent genotype (*rrn*A or AB/*vcg*E). These data were used to select a sub-set of 27 isolates for virulence testing with a subcutaneously inoculated, iron-dextran treated mouse model. Based on the mouse model data, 11 isolates were non-lethal, whereas 16 isolates were lethal, indicating a potential for human infection. Within the non-lethal group there were eight oyster and three clinical isolates. Six of the non-lethal isolates were the less virulent genotype (*rrn*A/*vcg*E or *rrn*AB/*vcg*E) and two were *rrn*B/*vcg*C with the remaining two of mixed genotype (*rrn*AB/*vcg*C and *rrn*B/*vcg*E). Of the lethal isolates, five were oysters and 11 were clinical. Eight of the lethal isolates were the less virulent genotype and seven the more virulent genotype, with the remaining isolate a mixed genotype (*rrn*A/*vcg*C). A discordance between virulence genotype and individual mouse virulence parameters (liver infection, skin infection, skin lesion score, and body temperature) was observed; the variable most strongly associated with mouse virulence parameters was season (warm or cold conditions at time of strain isolation), with more virulent strains isolated from cold conditions. These results indicate that biochemical profiles and genotype are not significantly associated with virulence potential, as determined by a mouse model. However, a relationship with virulence potential and seasonality was observed.

## Introduction

*Vibrio vulnificus* is a gram-negative opportunistic pathogen that is naturally found in shellfish and coastal brackish waters in warmer climates (Kaspar and Tamplin, 1993; Nilsson et al., 2003). It causes gastroenteritis, wound infections, or septicemia (Blake et al., 1979; Hlady and Klontz, 1996) through two primary routes: 1) consumption of raw shellfish, primarily oysters, and 2) exposure of open wounds to *V. vulnificus* (Shapiro et al., 1998; Oliver and Kaper, 2001). This bacterium is the deadliest foodborne pathogen with a case fatality rate greater than 30% (Hlady and Klontz, 1996; Shapiro et al., 1998; Scallan et al., 2011). Moreover, individuals who are immunocompromised have the greatest risk of mortality due to increased risk of sepsis (Strom and Paranjpye, 2000; Haq and Dayal, 2005). Although this pathogen has a low rate of infection (Strom and Paranjpye, 2000), it is important to investigate methods to evaluate the virulence potential of strains due to the severity of illness and high case fatality rate. Existing strategies to evaluate and mitigate the risks associated with *V. vulnificus* focus on the total population of the pathogen. However, there is evidence that not all strains have an equal potential to cause disease in humans, and identification of reliable markers of virulent populations would permit refinement of risk assessment models and mitigation efforts.

Biotyping and genetic markers are currently used to classify the virulence potential of *V. vulnificus*. There are three biotypes, defined by biochemical profiles, associated with virulence potential based on host specificity. Biotype 1 is associated with human infections (Warner and Oliver, 2008). Biotype 2 is associated with infections in eels and occasionally in humans (Veenstra et al., 1993; Amaro and Biosca, 1996). While biotype 3 has only been associated with wound infections of fish handlers (Bisharat et al., 1999). In addition, there are genetic markers used to subtype, primarily biotype 1, *V. vulnificus* isolates, which were developed based on source of strain isolation: clinical (*i.e.*, from an ill individual) or environmental (*e.g.*, shellfish, harvest water, etc.) (Nilsson et al., 2003). Rosche et al. (2005) identified two variants of the virulence correlated gene (*vcg*) which correlated with isolation source: clinical (*vcg*C) or environmental (*vcg*E). Additionally, an evaluation of the 16S rRNA gene (*rrn*) polymorphic variants identified two types, with *rrn*A primarily associated with environmental isolates and *rrn*B associated with clinical isolates (Nilsson et al., 2003; Vickery et al., 2007). The *vcg* and *rrn* genetic markers are often complementary to one another, with *rrn*B and *vcg*C most often identified in clinical isolates, and *rrn*A and *vcg*E genotypes appearing most often in environmental isolates (Han et al., 2009). Based on these strong associations between isolate source and gene variants, the genotype *rrn*A/*vcg*E is generally assumed to be less virulent, whereas *rrn*B/*vcg*C type strains are assumed to be more virulent (Jones et al., 2013). In addition, mannitol fermentation has been associated with the *rrn*B genotype (Drake et al., 2010), indicating this as a potential biochemical marker of virulence potential.

Subtyping and genotyping assays have served as a proxy for virulence potential based on the presumption that isolates from a clinical source are likely more virulent than environmental isolates, which has been largely supported in an animal model (Starks et al., 2000; DePaola et al., 2003). A subcutaneously (s.c.) inoculated iron-dextran treated mouse model has been used to evaluate virulence potential in *V. vulnificus* (Starks et al., 2000; DePaola et al., 2003; Thiaville et al., 2011). This model has revealed systemic infection and mortality presenting more often in mice injected with clinical strains (DePaola et al., 2003), while environmental strains appear to grow slower or are more easily attenuated by the mouse host (Starks et al., 2006). Thiaville et al. (2011) was one of the first studies that measured how virulence potential, as determined by the mouse model, relates to strain genotype on a large scale; however, the strain set selected for this study was somewhat limited, with oyster isolates from warmer months underrepresented. The study identified five virulence clusters associated with differing severity on type of mouse infection. Strains that caused systemic infection (liver) following skin infection were considered potentially lethal to humans, while less virulent strains (non-lethal) caused primarily skin infections. The study concluded that while *vcg*C was associated with virulence potential, it was not predictive of virulence in biotype 1 V*. vulnificus* strains (Thiaville et al., 2011). For example, some of the most virulent strains were of the *vcg*E genotype. Mouse models are not ideal assays due to resource and time requirements, as well as the ethical considerations; however, they remain the current gold standard for evaluating *V. vulnificus* virulence (Starks et al., 2000).

Previous studies have assumed that isolate source (*i.e*., clinical or environmental/food) is a reliable proxy for *V. vulnificus* virulence, but without specific testing they are intrinsically biased to that assumption. Investigations into the relationship of genotype and virulence potential, as measured in a mouse model, are limited by the number and diversity of the isolates examined. Most of the clinical isolates used in previous studies (Nilsson et al., 2003; DePaola et al., 2003; Vickery et al., 2007; Thiaville et al., 2011) were isolated in warm months (May–September), when most infections occur; however, the majority of environmental isolates for these studies were obtained from cooler months (October–April). Regardless, these studies indicate a relationship with (DePaola et al., 2003), but lack the predictive power to interpolate virulence potential from a common virulence genotypes (Thiaville et al., 2011). This raises questions about the utility of these existing typing schemes. Therefore, the current study aims to further investigate the relationship of genotype and virulence potential by utilizing a geographically and seasonally diverse set of *V. vulnificus* isolates. This isolate set was examined for partial biochemical profiles (API 20E), *rrn* and *vcg* genotypes, and virulence potential as determined through the s.c. inoculated iron-dextran treated mouse model, in order to determine if an association exists between virulence potential, biochemical phenotype, genotype, isolate source, and season of isolation.

## Materials and Methods

### *Vibrio vulnificus* Isolates Included in This Study

A total of 69 V*. vulnificus* isolates were selected for this study. All *V. vulnificus* were isolated in 2006–2007 from various parts of the United States. Of these, 30 were isolated from ill patients as part of the Cholera and Other Vibrio Illness Surveillance (COVIS) program and were contributed by the Centers for Disease Control and Prevention (**Table 1**) and 39 were isolated from retail level raw oysters [**Table 2**; (DePaola et al., 2010)]. All isolates were purified and confirmed as *V. vulnificus* by real-time PCR as previously described (Kinsey et al., 2015). Isolates were stored in TSB + 30% glycerol at −80°C.

**Table 1:** Clinical *Vibrio vulnificus* isolates.

Isolate ID	Month of Isolation	Reporting Date	Reporting State	Source
CDC_K4567	Jul	2006	HI	CLINICAL; OTHER
CDC_K4574	Nov	2006	HI	BLOOD
CDC_K4633	Dec	2006	OH	BLOOD
CDC_K4712	unknown	Unknown	RI	CLINICAL; UNKNOWN
CDC_K4767	Aug	2006	VA	BLOOD
CDC_K4776	Jul	2006	AL	CLINICAL; OTHER
CDC_K5008	Apr	2007	MS	BLOOD
CDC_K5041	Apr	2007	TX	BLOOD
CDC_K5056	May	2007	TX	BLOOD
CDC_K5057	May	2007	TX	BLOOD
CDC_K5060	May	2007	GA	BLOOD
CDC_K5148	Jun	2007	MS	BLOOD
CDC_K5204-LT	Jun	2007	TX	BLOOD
CDC_K5287*	May	2007	HI	BLOOD
CDC_K5326	May	2007	VA	BLOOD
CDC_K5616	Jul	2007	NY	BLOOD
CDC_K5327	Jul	2007	VA	CLINICAL; OTHER
CDC_K5333	May	2007	TX	CLINICAL; OTHER
CDC_K5338	Aug	2007	GA	BLOOD
CDC_K5486	Jul	2007	NC	CLINICAL; OTHER
CDC_K5583	Oct	2007	GA	BLOOD
CDC_K5585	Nov	2007	GA	BLOOD
CDC_07-2405	Oct	2006	LA	STOOL
CDC_07-2418	Mar	2007	LA	BLOOD
CDC_07-2444	Sept	2007	IL	BLOOD
CDC_K4572*	Oct	2006	HI	BLOOD
CDC_K4778*	Sept	2006	AL	CLINICAL; OTHER
CDC_K5613*	Jul	2007	NY	BLOOD
CDC_K5636*	unknown	Unknown	MD	BLOOD
CDC_K5637*	Sept	2007	MD	BLOOD

*Was not identified as V. vulnificus with API 20E.

All isolates were provided by the Centers for Disease Control and Prevention. Reporting information (month/year and state reporting) was obtained from COVIS forms. The reporting state is the state from which the isolate was received and not necessarily the state from which the infection was contracted.

**Table 2:** Oyster *Vibrio vulnificus* isolates.

Isolate ID	Month of Isolation	Date of Harvest	State of Harvest
FDA_R101-A9	Nov	2007	AL
FDA_R101-D8	Nov	2007	AL
FDA_R11-B3	Feb	2007	LA
FDA_R19-C1	Mar	2007	TX
FDA_R27-C9	Apr	2007	LA
FDA_R30-C10	Apr	2007	FL
FDA_R42-D10	May	2007	LA
FDA_R47-E7	Jun	2007	TX
FDA_R499-A8	May	2007	VA
FDA_R51-A12	Jun	2007	LA
FDA_R51-E9	Jun	2007	LA
FDA_R53-A6	Jul	2007	FL
FDA_R57-B10	Jul	2007	LA
FDA_R595-A3	Jul	2007	LA
FDA_R595-D7	Jul	2007	LA
FDA_R595-D8	Jul	2007	LA
FDA_R59-B3	Jul	2007	AL
FDA_R63-A4	Jul	2007	CT
FDA_R63-C5	Jul	2007	LA
FDA_R73-C11	Sept	2007	DE
FDA_R74-C3	Sept	2007	LA
FDA_R74-D6	Sept	2007	LA
FDA_R80-G3	Sept	2007	LA
FDA_R80-G5	Sept	2007	LA
FDA_R80-H6	Sept	2007	LA
FDA_R81-C6	Sept	2007	LA
FDA_R81-F5	Sept	2007	LA
FDA_R844-G9	Aug	2007	FL
FDA_R84-F1	Sept	2007	VA
FDA_R85-B11	Oct	2007	SC
FDA_R96-B9	Oct	2007	DE
FDA_R97-A5	Oct	2007	VA
FDA_R97-B5	Oct	2007	VA
FDA_R98-C1	Oct	2007	LA
FDA_R98-C11	Oct	2007	LA
FDA_R98-E6	Oct	2007	LA
FDA_R99-A10	Oct	2007	LA
FDA_R844-F10*	Aug	2007	FL
FDA_R60-F9*	Jul	2007	NJ

*Was not identified as V. vulnificus with API 20E.

All isolates were obtained from retail oysters as described in DePaola et al., 2010. Harvest information was obtained from shellfish tags collected with the oyster samples.

### Biochemical Characterization of Isolates

To evaluate partial biochemical profiles of the *V. vulnificus* isolates, API 20E (BioMerieux, Durham, NC) test strips were used according to the manufacturer’s protocol, except that 2% NaCl was used for cell suspensions (Kaysner et al., 2004; Martinez-Urtaza et al., 2005). Oxidase tests were completed using Dry Slides (BBL, Difco, Sparks, MD). API 20E results were entered into the manufacturer’s database for identification.

### Determination of *vcg* and *rrn* Genotypes

Isolates were streaked from frozen stocks to Tryptic Soy Agar (TSA; Difco) to confirm purity. A single colony was transferred to Tryptic Soy Broth (TSB; Remel, Atlanta, GA) and incubated at 35 ± 2°C for 18–24 h. One ml of the overnight culture was transferred to a microfuge tube and heated at 95–100°C for 10 min to produce a crude DNA lysate, which was used as template in subsequent real-time PCR assays. Isolate genotypes were determined using qualitative real-time PCR assays as previously described for 16S rRNA (*rrn*) gene type A or B (Vickery et al., 2007) and virulence correlated gene (*vcg*) type C or E (Drake et al., 2010) on a SmartCycler II System (Cepheid, Sunnyvale, CA). Results were used to define more (*rrn*B and *vcg*C) or less (*rrn*A or *rrn*AB and *vcg*E) virulent genotype categories.

### Virulence Testing With Mouse Model

For mouse virulence testing, 27 isolates were selected to be representative of source and genotype combinations. Approximately 1,000 CFU of each strain was inoculated into at least two groups of five mice, as previously described (Thiaville et al., 2011). Rectal temperature was used as an indicator of illness severity and as a surrogate for death (<33°C was determined to be dead) prior to sacrifice, when animals survived. Colony forming units (CFUs) were determined by standard plate count from the skin and liver following homogenization to determine local and systemic infection, respectively. Skin lesions were scored based on the size and nature of the lesion using a scale of 1–4. The skin and liver CFU data were used to cluster strains into virulence groups as previously described (Thiaville et al., 2011): Group 1 strains caused low skin and undetectable liver (systemic) infection, Group 2 strains caused moderate skin infection with little to no liver infection, Group 3 strains caused a high skin infection but low liver infection, Group 4 strains caused high skin and moderate liver infection, and Group 5 strains caused very high skin and very high liver infection. Assuming mouse virulence translates to human infection, Group 1, 2, and 3 strains would likely not be able to cause lethal infection in humans. Group 4 and 5 strains, because they cause high skin and moderate to high liver infection, have the potential to cause lethal infection in humans.

### Statistical Analyses

Data were analyzed by strain, using the mean results from all mice inoculated with that strain as the data point for each measured mouse virulence parameter to capture strain variability rather than individual mouse response. A three factor ANOVA (General Linear Model; GLM) was used to determine if interactions between source of isolation (clinical/oyster), genotype (more virulent/less virulent), or season of isolation (warm/cool) existed. As no significant interactions were found, GLM was used to evaluate quantitative data (log CFU/g skin, log CFU/g liver, body temperature, skin lesion score, and mortality) in comparison to the fixed variables. Fisher’s exact test was used to evaluate associations between isolate source, genotype, and season of isolation with biochemical reactions and lethal or non-lethal designation. Season of isolation was determined by grouping strains as either cool (October–April) or warm (May–September) seasons when *V. vulnificus* is historically less or more prevalent, respectively. Due to mixed genotype results, three isolates (FDA_R101-A9, CDC_K5148, and CDC_07-2444) were removed from statistical analyses in genotype comparisons. All statistical comparisons were conducted in JMP 13.

## Results

### Biochemical Profiles of *Vibrio vulnificus* Isolates

API 20E identified 61 isolates as *V. vulnificus*, with the eight remaining isolates identified as *Vibrio* spp. or *Aeromonas* spp. (**Tables 1** **and** **2**). As API 20E may incorrectly identify *Vibrio* spp. (O’Hara et al., 2003; Sanjuan et al., 2009), PCR targeting the *vvh* gene (Kinsey et al., 2015), was used to confirm all 69 isolates as *V. vulnificus*. All *V. vulnificus* isolates (n = 69) were positive for fermentation of glucose and amygdalin and the presence of oxidase and negative for arginine dihydrolase, tryptophan deaminase, inositol fermentation, sorbitol fermentation, rhamnose fermentation, and arabinose fermentation (**Table 3**).

**Table 3:** Biochemical properties of *V. vulnificus* isolates.

	Test result (% of isolates) ^a^	
Characteristic	Clinical Isolates	Oyster Isolates
Oxidase	+ (100)	+ (100)
Amygdalin fermentation	+ (100)	+ (100)
Glucose fermentation	+ (100)	+ (100)
Lysine decarboxylase	+ (100)	+ (97)
*β*-Galactosidase	+ (97)	+ (97)
Gelatinase	+ (97)	+ (95)
Indole production	+ (100)	V (54)
Mannitol fermentation	V (57)	V (39)
Ornithine decarboxylase	− (97)	V (74)
Citrate utilization	− (100)	V (46)
Saccharose fermentation	− (83)	− (95)
Voges-Proskauer reaction (acetoin production)	− (100)	− (92)
H_2_S production	− (100)	− (97)
Urease	− (100)	− (97)
Melibiose fermentation	− (100)	− (97)
Arginine dihydrolase	− (100)	− (100)
Tryptophan deaminase	− (100)	− (100)
Inositol fermentation	− (100)	− (100)
Sorbitol fermentation	− (100)	− (100)
Rhamnose fermentation	− (100)	− (100)
Arabinose fermentation	− (100)	− (100)

a + if >80% positive, − if >80% negative. V, variable positive percent reported.

Additionally, some of the tests were generally positive (*β*-Galactosidase, lysine decarboxylase, and gelatinase), while others were generally negative (H_2_S production, urease, Voges–Proskauer reaction, saccharose fermentation, and melibiose fermentation). This data may be useful in biochemical identification of *V. vulnificus*. Interestingly, there were three biochemical tests (ornithine decarboxylase, citrate utilization, and indole production) which were variable in oyster isolates, but generally present in clinical isolates, resulting in a statistically significant association between isolate source and these biochemical tests (p < 0.04). In this study, mannitol fermentation, which has been associated with virulent genotypes of *V. vulnificus* (Drake et al., 2010), was also significantly associated with the virulent genotypes (p < 0.001) here. Mannitol was the only biochemical reaction that was variable in both clinical and oyster isolates: 57% of clinical and 39% of oyster isolates were positive (**Table 3**).

### Virulence Genotyping of *Vibrio vulnificus* Isolates

For virulence genotyping of the clinical isolates (n = 30), 53% were the less virulent *rrn*A or AB/*vcg*E, and 47% were the more virulent (*rrn*B/*vcg*C) genotype (**Table 4**). Additionally, two clinical isolates had mixed genotypes: CDC_K5148 (*rrn*B/*vcg*E) and CDC_07-2444 (*rrn*A/*vcg*C). Of the strains isolated from blood infections (n = 22), half were the less virulent genotype and half the more virulent genotype. In the subset (n = 8) of clinical isolates not isolated from blood, half were the less virulent genotype, three were the more virulent genotype, and one was a mixed genotype (data not shown). Oyster isolate (n = 39) genotyping identified 64% of isolates as the less virulent genotype, *rrn*A or *rrn*AB/*vcg*E, and with a mixed genotype: FDA_R101-A9 (*rrn*AB/*vcg*C) and FDA_R63-C5 (*rrn*B/*vcg*E). The remaining oyster isolates (33%) were the more virulent (*rrn*B/*vcg*C) genotype.

**Table 4:** Genotype of *V. vulnificus* strains.

Origin		*vcg*C	*vcg*E	Total
	*rrn*A	1	15	16
*Clinical*	*rrn*AB	–	–	–
	*rrn*B	13	1	14
	*rrn*A	–	22	22
*Oyster*	*rrn*AB	–	3	3
	*rrn*B	13	1	14
	Total	28	41	69

### Virulence of *Vibrio vulnificus* Isolates in the Mouse Model

None of the *V. vulnificus* isolates in this study fell into mouse virulence Group 1, the least virulent group. Six isolates (22%) were classified as Group 2 (**Table 5**), with moderate skin infection (6.3–7.7 log CFU/g) and little to no liver infection (1.4–3.3 log CFU/g): five oyster and one clinical; five *rrn*A/*vcg*E and one mixed genotype (*rrn*AB/*vcg*E). Only one of these isolates, FDA_R51-A12 (*rrn*A/*vcg*E), caused mortality and all skin lesion scores were <3. Four isolates (15%) fell into Group 3: three oyster and one clinical; one *rrn*A/*vcg*E and three *rrn*B/*vcg*C. An additional clinical isolate, CDC_K5148 (*rrn*B/*vcg*E), was in its own novel classification, Group 6. However, based on its general characteristics, it is similar to Group 3, with high skin infection (7.8–8.5 log CFU/g) and low liver infection (2.8–3.7 log CFU/g for the Group 3 isolates and 1.4 log CFU/g for CDC_K5148). Only one of the Group 3 isolates, FDA_R73-C11 (*rrn*A/*vcg*E), caused mortality. All skin lesion scores were 3–4. Over half (56%) of the *V. vulnificus* isolates fell into Group 4: 5 oyster and 10 clinical; 8 *rrn*A or *rrn*AB/*vcg*E, 6 *rrn*B/*vcg*C, and 1 *rrn*A/*vcg*C. This group had high skin infection (7.2–8.3 log CFU/g) and moderate liver infection (4.4–6.0 log CFU/g). All strains caused mortality, ranging from 10% (FDA_R595-D7, *rrn*AB/vcgE) to 80% (CDC_07-2444, *rrn*A/vcgC). Skin lesion score for this group ranged from 2.8 to 4. One isolate, FDA_K5338 (*rrn*B/vcgC), was characterized as Group 5, with very high skin (8.5 log CFU/g) and very high liver (6.7 log CFU/g) infection.

**Table 5:** Mouse virulence data. Skin infection, liver infection, body temperature, and skin lesion data are provided as means of the 5–10 animals tested with each strain.

Strain	Genotype	Log_10_ Skin CFU/g	Log_10_ Liver CFU/g	Temp (°C)	Skin Lesion Score	Mortality	Virulence Group	Virulence Potential
CDC_K4574	*rrn*A/vcgE	6.8	1.4	38.2	2.2	0%	2	Non-lethal
FDA_R51-A12	*rrn*A/vcgE	7.5	3.3	34.3	2.8	7%	2	
FDA_R84-F1	*rrn*A/vcgE	6.3	1.5	37.5	1.2	0%	2	
FDA_R499-A8	*rrn*A/vcgE	7.7	1.9	37.8	1.8	0%	2	
FDA_R844-G9	*rrn*A/vcgE	6.3	1.7	38.1	2.2	0%	2	
FDA_R101-A9	*rrn*AB/vcgC	6.5	2.1	35.1	1.4	0%	2	
CDC_K5613	*rrn*B/vcgC	7.8	3.3	36.7	3.4	0%	3	
FDA_R73-C11	*rrn*A/vcgE	8.3	3.7	37.0	3.4	20%	3	
FDA_R63-A4	*rrn*B/vcgC	7.9	3.1	33.3	4.0	0%	3	
FDA_R63-C5	*rrn*B/vcgC	8.5	2.8	35.8	3.4	0%	3	
CDC_K5148	*rrn*B/vcgE	8.5	1.4	38.4	3.0	0%	6*	
CDC_K4767	*rrn*A/vcgE	7.5	5.0	32.8	3.0	30%	4	Lethal
CDC_K5008	*rrn*A/vcgE	7.9	4.6	31.7	4.0	60%	4	
CDC_K5326	*rrn*A/vcgE	7.7	4.9	31.5	4.0	40%	4	
CDC_K5583	*rrn*A/vcgE	8.1	4.5	33.4	4.0	20%	4	
CDC_07-2444	*rrn*A/vcgC	7.2	5.4	30.5	4.0	80%	4	
CDC_K4633	*rrn*B/vcgC	7.8	5.8	31.3	4.0	50%	4	
CDC_K4776	*rrn*B/vcgC	7.4	4.3	33.4	2.8	67%	4	
CDC_K5041	*rrn*B/vcgC	8.0	5.1	32.0	3.6	40%	4	
CDC_K5204-LT	*rrn*B/vcgC	8.1	4.4	31.5	3.4	50%	4	
CDC_07-2405	*rrn*B/vcgC	8.3	5.7	31.2	3.2	60%	4	
FDA_R19-C1	*rrn*A/vcgE	7.9	6.0	31.6	4.0	20%	4	
FDA_R27-C9	*rrn*A/vcgE	7.4	4.8	33.2	3.2	20%	4	
FDA_R98-C1	*rrn*A/vcgE	8.0	5.9	31.4	4.0	30%	4	
FDA_R595-D7	*rrn*AB/vcgE	8.1	5.1	32.6	3.6	10%	4	
FDA_R98-E6	*rrn*B/vcgC	8.0	5.4	32.2	4.0	30%	4	
CDC_K5338	*rrn*B/vcgC	8.5	6.7	31.6	4.0	90%	5	

*Virulence Group 6 is a novel group identified in this study and is categorized as non-lethal.

Percent mortality is calculated based on the group of mice.

### Association of Mouse Virulence With Isolate Source, Virulence Genotype, and Season of Isolation

Evaluation of the association between isolate source (clinical or oyster), virulence genotype (more virulent or less virulent), and season of strain isolation (cold or warm) with mouse virulence parameters including skin infection (log CFU/g skin), liver infection (log CFU/g liver), mouse temperature (as a proxy for illness severity), and mouse mortality (percentage of mice tested that died) were examined. The majority (6 of 11) of isolates with very high skin infection (≥8 log CFU/g) in the mouse model were the more virulent genotype or were of clinical origin. Similarly, isolates with very high liver (≥5 log CFU/g) infection were the more virulent genotype (five of 10) or were of clinical origin (six of 10).

**Figure 1:**
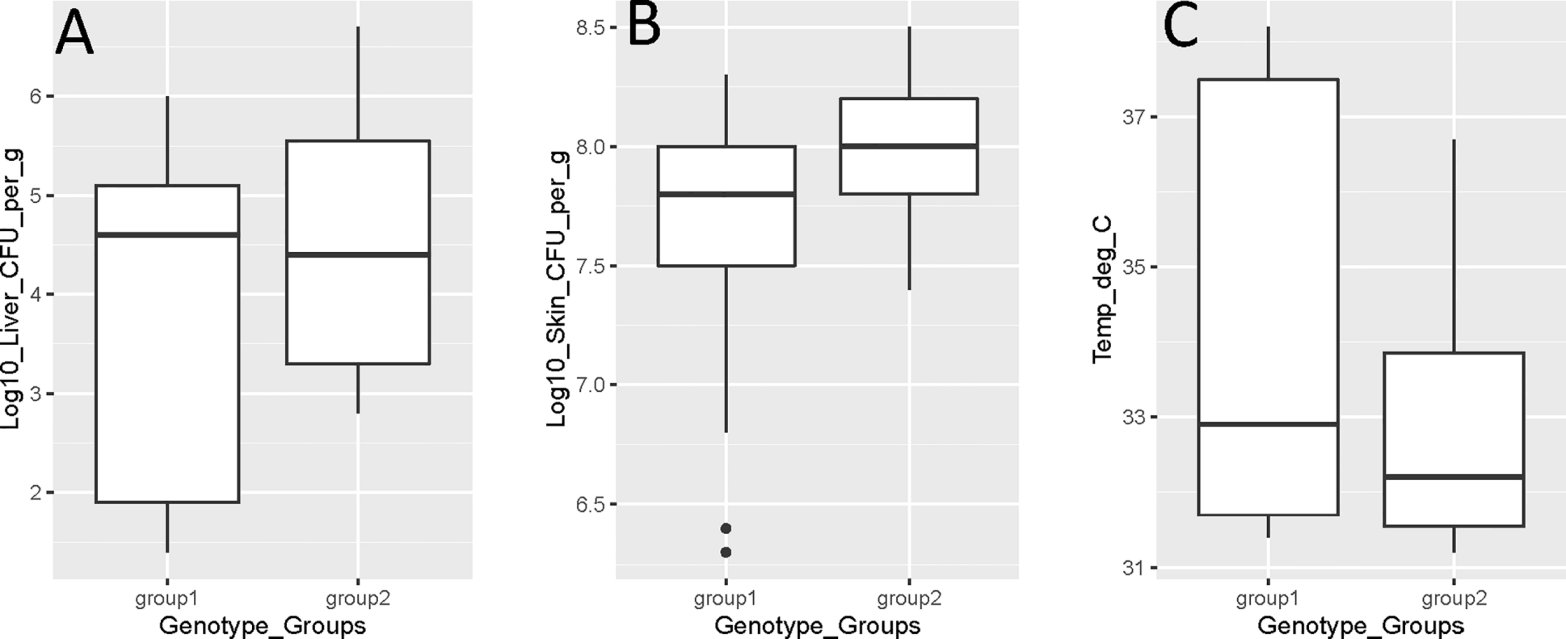
*Vibrio vulnificus* mouse model virulence parameter data [**(A)** liver infection; **(B)** skin infection; **(C)** mouse temperature] by genotype (group 1 is *rrn*A/*vcg*E and *rrn*AB/*vcg*E; group 2 is *rrn*B/*vcg*C). Each box displays the median with the upper (25%) and lower (75%) quartiles as hinges. Whiskers represent the highest or lowest observation + 1.5*Inter quartile range. Dots represent outliers.

However, there were no strong statistically significant relationships (p > 0.05) between the virulence genotype and skin infection, liver infection, mouse temperature, or mouse mortality (**Figure 1**). However, there was a weak association with virulence genotype and skin infection (p = 0.05). Similarly, no statistically significant (p > 0.05) relationships between isolate source and skin infection, liver infection, or mouse temperature were identified. There was a strong association between isolate source and mouse mortality, with clinical isolates causing significantly more mortality than oyster isolates (p = 0.003). Isolate source was also weakly associated with the lethal *versus* non-lethal categorization of mouse data, with clinical isolates being significantly more lethal (p = 0.05) than oyster isolates.

**Figure 2:**
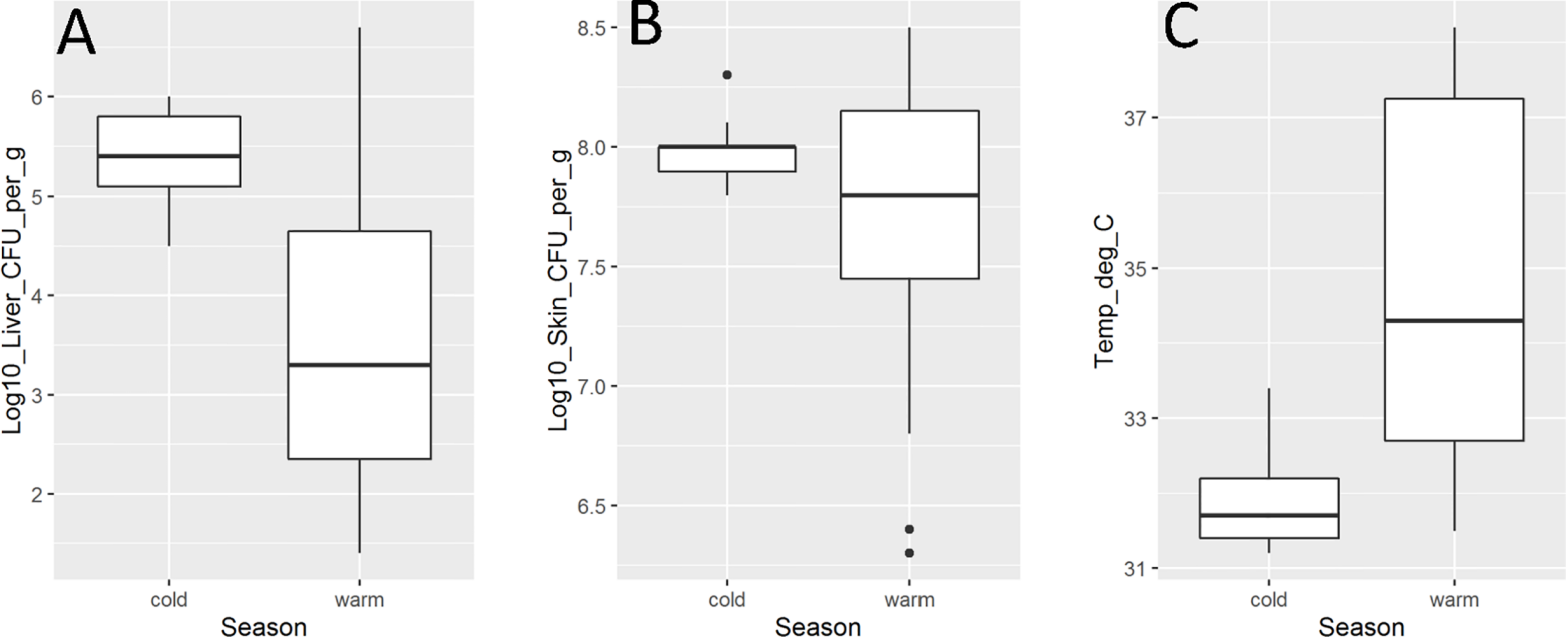
*Vibrio vulnificus* mouse model virulence parameter data [**(A)**. liver infection; **(B)**. skin infection; **(C)**. mouse temperature] by season (cold is October–April; warm is May–September). Isolate from HI (CDC_K4574) is grouped with warm season due to lack of temperature variability in the state. Each box displays the median with the upper (25%) and lower (75%) quartiles as hinges. Whiskers represent the highest or lowest observation + 1.5*Inter quartile range. Dots represent outliers.

The extrinsic factor with the strongest association to mouse virulence of *V. vulnificus* strains was season of isolation with significant associations between season of isolation and liver infection (p = 0.04) and mouse temperature (p = 0.04) identified (**Figure 2**). In both cases, isolates from the cooler season were more virulent, *i.e.*, caused higher liver infection and lower mouse body temperatures. There were no statistically significant relationships (p > 0.05) between season of isolation and skin infection or mouse mortality.

## Discussion

### Biochemical Profiles of *Vibrio vulnificus* Isolates

Biochemical profiles were determined by API 20E. Only 88% of the *V. vulnificus* isolates were correctly identified, which is slightly higher than previous reports (O’Hara et al., 2003). This is likely due to the use of 2% NaCl for inoculation of the biochemical test, rather than the manufacturer’s recommended 0.85%, as previously described as an improved identification method for *Vibrio* spp. from the environment (Martinez-Urtaza et al., 2005). Interestingly, a higher rate of misidentification was noted for the clinical isolates (20%), as compared to the oyster isolates (5%), which is contrary to previous findings (Martinez-Urtaza et al., 2005). Another noteworthy observation was the significantly (p = 0.02) lower presence of indole production in isolates from the cooler season. A higher variability in biochemical profiles was observed for the oyster isolates as compared to the clinical isolates. This difference in variability has not been noted previously, but is logical assuming environmental (oyster) isolates are under less selective pressure than clinical isolates, which need specific traits to survive in the human host.

### Genotyping of *Vibrio vulnificus* Isolates

In studies establishing and investigating the utility of *V. vulnificus* genotypes for association with virulence potential (Nilsson et al., 2003; DePaola et al., 2003; Vickery et al., 2007; Thiaville et al., 2011) a similar set of isolates was used. Repeated use of this set of *V. vulnificus* isolates provided good reference for repeatability and is valuable for assay development. However, the isolate set remains limited in that clinical isolates were from the warm months (May–September) and environmental isolates were from cooler months (October–April). In contrast, this study utilized a more balanced set of isolates collected from a range of seasonal and geographical sources. As a result, well-defined relationships between isolate origin and genotype were not clearly identified.

While the majority (64%) of oyster isolates were *rrn*A/*vcg*E, the prevalence of the *rrn*B/*vcg*C genotype (33%) was higher than a previous observation where virulent genotypes represented ~6% of isolates from the environment (Rosche et al., 2005). However, recent studies found results similar to the current work, with reports of up to 40% of *V. vulnificus* isolated from oysters having the virulent genotype (Han et al., 2009; Drake et al., 2010; Jones et al., 2013). As *V. vulnificus* that causes infection originates from the environment (where exposure occurs), one would expect a mix of genotypes and virulence potential such as observed in the current work.

Genotypes of clinical *V. vulnificus* isolates in this study were surprising, with a lower than expected prevalence of the *rrn*B/*vcg*C genotype. This holds true even when looking at the subset of clinical isolates from blood cultures and deviates from previous findings where isolates of clinical origin are nearly all *rrn*B/*vcg*C (Nilsson et al., 2003; Warner and Oliver, 2008; Drake et al., 2010). We hypothesized this discrepancy is due to seasonal and geographic variability, as our study focused on a more diverse panel compared to previous studies. This theory is supported by previous studies that observed differences in distribution of genotypes in the environment based on season and/or region (Lin and Schwarz, 2003; Warner and Oliver, 2008; Jones et al., 2013; Williams et al., 2017).

### Association of Mouse Virulence With Biochemical Phenotype, Virulence Genotype, and Season of Isolation

Mouse virulence testing has previously identified non-lethal (Groups 1–3) and lethal (Groups 4–5) clusters (Thiaville et al., 2011). When using this model, with a greater sensitivity for liver infection, a novel, non-lethal cluster (Group 6) was identified. This Group caused high rates of skin infection, but low liver infection, so was classified as non-lethal. One surprising finding was that no isolates fell into virulence Group 1 (the least virulent group), even with a diversity of oyster isolates tested. Although this is different from the findings of Thiaville et al. (2011), where 16% of isolates were Group 1, the two studies are similar in that the majority of isolates were identified as Group 4 in both. Taken together, these findings suggest that the majority of *V. vulnificus* isolates can cause high skin and moderate liver infection, regardless of genotype or isolation source.

We found that lethal and non-lethal strains were only weakly correlated to clinical or oyster origin of the isolates, but strongly correlated to mouse mortality. Regardless of statistical associations, the non-lethal isolates were generally from oysters and have the less virulent genotype, as expected. Additionally, all strains that resulted in >40% mouse mortality were isolated from a clinical source, and all but one had the more virulent genotype. These observations, along with the previous data used in establishing the iron-dextran mouse model (Starks et al., 2000; DePaola et al., 2003), support the utility of this model and validity of resultant data. Interestingly, no correlation was identified between the virulence genotype of isolates and the observed mouse virulence parameters, other than skin infection. These genotypes have been reported as an indicator of severe illness potential in humans; however, the lack of correlation with mouse virulence (as a proxy for potential human infection) questions their suitability as predictors. These results are consistent with previous research demonstrating that genotypes are associated with, but do not predict, virulence in a mouse model (Thiaville et al., 2011).

Season of isolation was the factor which most correlated with virulence potential in *V. vulnificus*. Isolates from the cooler months (except the isolate from Hawaii) were lethal and were associated with higher levels of liver infection and lower body temperature. Isolates from the warmer season had mixed virulence potential; this is reflected by the wide range in liver and skin infection, as well as body temperature. Environmental drivers of *Vibrio* spp. are often attributed to two main factors, temperature and salinity, the primary differences across seasons, with higher levels generally found during the warmer season (Takemura et al., 2014; Johnson, 2015). Our results, therefore, may appear counter-intuitive, especially combined with knowledge that the majority of *V. vulnificus* infections occur during warmer months (Oliver, 2015; Centers for Disease Control and Prevention, 2017). However, this apparent discrepancy may be explained by a higher proportion of virulent *V. vulnificus* in the environment (and oysters) during the cooler season when total populations are lowest, similar to what has been observed for *V. parahaemolyticus* (Johnson et al., 2010; DePaola et al., 2010). The hypothesis that a greater proportion of the *V. vulnificus* population is virulent during the cooler season is supported by objective evidence of *V. vulnificus* infections from oyster consumption in the US (as reported to the FDA). During the cooler months (November–April), 10% of oysters have >3 log *V. vulnificus*/g compared to 34% of oysters with these high levels of *V. vulnificus* during the warm months (DePaola et al., 2010). Assuming equally virulent populations, it would be expected that there be three times more cases in the warmer months than the colder months; however, the difference is not always that large (Personal Communication (2020)), suggesting a higher proportion of disease-causing strains in the cooler season. Taken together, these data indicate an association between virulence and season; additionally, this is the first report to define this association using mouse model data.

### Conclusions and Future Directions

Due to the lack of reliable markers for virulence potential, current risk evaluation and management strategies are based on total *V. vulnificus* populations. This study indicates that *vcg*C and *rrn*B, two gene variant candidates for differentiating isolate virulence, are not reliable markers of systemic virulence potential in *V. vulnificus*. It is likely that the different genotypes are reflective of a bifurcation of phylogenetic lineage (Lopez-Perez et al., 2019), rather than functional differences. This, and previous studies, demonstrated that although no reliable indicator, or set of indicators, has yet been identified, not all *V. vulnificus* isolates have the same virulence potential. In addition, we have identified a relationship between isolates from the cooler season and systemic virulence potential in *V. vulnificus*.

Using the findings of this work as a basis, future research may be directed towards identifying markers to differentiate virulence potential and to identify the driving factor(s) behind the association between season and virulence. Identification of those factors may allow focus on regulatory pathways such as long-term cold adaptation of *V. vulnificus*. Next-generation sequencing makes it is possible to discern new potential markers and pathways important to *V. vulnificus* virulence with isolate virulence known *a priori*. Previous SOLiD sequencing of four *V. vulnificus* isolates (Gulig et al., 2010), resulted in an extensive list of potential markers, likely due a lack of robust coverage of isolate genomes and small sample size. By sequencing additional well-characterized isolates on platforms with increased genome coverage, we can more readily narrow down these lists of candidate virulence genes. Identification of reliable virulence markers would allow for risk assessments and risk management approaches to be refined in order to better protect public health.

## Data Availability Statement

The original contributions presented in the study are included in the article/supplementary material. Further inquiries can be directed to the corresponding author.

## Ethics Statement

The animal study was reviewed and approved by the Institutional Animal Care & Use Committee, PO Box 100142 Gainesville, Florida 32610-0142, Chair, Michael Katovich, University of Florida.

## Author Contributions

Designed experiment: JJ. Conducted experiments: TK, JJ, CL, and PG. Wrote paper: KL and JJ. Edited paper: TK, CL, and PG. Analysis: JJ, KL, TK, CL, and PG. All authors contributed to the article and approved the submitted version.

## Conflict of Interest

The authors declare that the research was conducted in the absence of any commercial or financial relationships that could be construed as a potential conflict of interest.
